# Mutation based treatment recommendations from next generation sequencing data: a comparison of web tools

**DOI:** 10.18632/oncotarget.8017

**Published:** 2016-03-09

**Authors:** Jaymin M. Patel, Joshua Knopf, Eric Reiner, Veerle Bossuyt, Lianne Epstein, Michael DiGiovanna, Gina Chung, Andrea Silber, Tara Sanft, Erin Hofstatter, Sarah Mougalian, Maysa Abu-Khalaf, James Platt, Weiwei Shi, Peter Gershkovich, Christos Hatzis, Lajos Pusztai

**Affiliations:** ^1^ Medical Oncology, Yale Cancer Center, Yale School of Medicine, CT 06520, New Haven, USA; ^2^ Pathology, Yale Cancer Center, Yale School of Medicine, CT 06520, New Haven, USA; ^3^ Radiology, Yale Cancer Center, Yale School of Medicine, CT 06520, New Haven, USA

**Keywords:** breast cancer, biomarkers and intervention studies, mutation based treatment recommendations, tumor profiling, personalized medicine

## Abstract

Interpretation of complex cancer genome data, generated by tumor target profiling platforms, is key for the success of personalized cancer therapy. How to draw therapeutic conclusions from tumor profiling results is not standardized and may vary among commercial and academically-affiliated recommendation tools. We performed targeted sequencing of 315 genes from 75 metastatic breast cancer biopsies using the FoundationOne assay. Results were run through 4 different web tools including the Drug-Gene Interaction Database (DGidb), My Cancer Genome (MCG), Personalized Cancer Therapy (PCT), and cBioPortal, for drug and clinical trial recommendations. These recommendations were compared amongst each other and to those provided by FoundationOne. The identification of a gene as targetable varied across the different recommendation sources. Only 33% of cases had 4 or more sources recommend the same drug for at least one of the usually several altered genes found in tumor biopsies. These results indicate further development and standardization of broadly applicable software tools that assist in our therapeutic interpretation of genomic data is needed. Existing algorithms for data acquisition, integration and interpretation will likely need to incorporate artificial intelligence tools to improve both content and real-time status.

## INTRODUCTION

Molecular target profiling of cancer is readily available in the clinic through commercial diagnostic companies and CLIA-accredited academic laboratories. Many of the assays rely on next generation sequencing methods, sometimes also including other techniques, to detect DNA sequence changes or other molecular abnormalities in tumor specimens. It is expected that the results will assist in selecting a therapy that is tailored to the specific molecular abnormalities of a given cancer [1]. Guidelines exist for the analytical validation of next generation sequencing and omics-based assays [2, 3] and the technical reliability of several clinically available assays have been published [4–6]. The clinical interpretation of the results, however, is less clear.

Therapeutic interpretation of genomic data from tumor biopsies is challenging at multiple levels. It has been suggested that molecular alterations in a single biopsy may not be representative of the entire tumor [7]. However, it is also clear that the majority of molecular abnormalities are shared across different regions of a cancer and even between metastatic sites, therefore the importance of private mutations is still debated. Most commercial, and many academic laboratories, perform tumor-only sequencing and it is increasingly recognized that several of the variants (up to 15–20%) that are assumed to be somatic mutations may in fact be germline alterations. To what extent germline variants represent actionable targets is unknown [8]. Furthermore, there is no perfect method to predict the impact of a nucleic acid variant on protein function. Many computational algorithms exist that predict functional impact for a variant (SIFT, PolyPhen, Mutation Assessor, etc) but the true accuracy of these predictions is uncertain and they often yield conflicting results for the same variant [9]. Laboratory validation of biological impact is only available for very few mutations and variants. Finally, linking molecular abnormalities to potential therapeutic agents is also challenging and often inconsistent [10].

Putting mutations into a therapeutic context requires a broad range of expertise and a substantial amount of time on literature and database searches. Short of the handful of clinically validated targets (e.g. EGFR, c-KIT, HER- 2, AKT, ROS1, BRAF, BCR-ABL, PML-RARα) that represent only a very small subset of mutated genes, there is no universal agreement, or algorithm, for determining what mutations in what genes are clinically actionable and what treatment to recommend. Many academic institutions have started molecular tumor boards which include clinical disease experts, molecular pathologists, bioinformaticians, laboratory scientists and phase I clinical trialists to assist in the clinical interpretation of complex genomic data [11, 12]. Several academic institutions and scientific organizations have developed free online tools to assist this process. The Drug-Gene Interaction Database [13], My Cancer Genome [14], Personalized Cancer Therapy [15] and cBioPortal [16–18] represent the most readily available public resources to match genes and particular mutations to therapies that might target these. Each of these tools employs a distinct strategy that includes various combinations of automatic search algorithms and manual curation to synthesize information from databases and from the published literature.

The purpose of this study was to examine to what extent the different web tools identify similar therapeutic options for a given set of gene level anomalies observed in a cancer biopsy and if these options are concordant with those suggested by the diagnostic laboratory that has performed the molecular profiling.

## RESULTS

One hundred and three genes had predicted deleterious nucleic acid variants or copy number changes in at least one of the biopsy specimens determined by a commercial diagnostic laboratory; we refer to these as “mutated genes”. The median number of mutated genes per specimen was 5 (range 2 to 18). The most commonly affected gene was TP53, 37 of 75 (49%) specimens had alterations, followed by the PIK3CA (40%). Table 1 lists each of the affected genes and their frequencies. Forty three genes (42%) had at least one FDA-approved drug recommendation by at least one tool (Table 3). The website that identified the most drugs was DGIdb (for 36 genes) followed by the FoundationOne report (for 31 genes), cBioPortal (for 25 genes), MCG (10 genes) and PCT (for 9 genes). The most commonly recommended drugs were temsirolimus for alterations in 15 genes (PIK3CA, PIK3R1, PTEN, STK11, AKT-1, -2, -3, TSC2, KIT, PDGFRB, KRAS, VHL, RPTOR, NF1, BRAF) followed by everolimus for 14 genes (same as temsirolimus except BRAF) and regorafenib for 10 genes (FGFR -1, -2, FLT -2, - 4, KDR, KIT, KRAS, PDGFRβ, VHL, BRAF) (Table 3).

**Table 1:** Mutations from 75 advanced breast cancer cases in order of frequency

Gene	% Cases	# Cases	Gene	% Cases	# Cases	Gene	% Cases	# Cases
**TP53**	49.33%	37	**ERBB3**	4.00%	3	CARD11	1.33%	1
**PIK3CA**	40.00%	30	LRP1B	4.00%	3	CDK4	1.33%	1
**CCND1**	30.67%	23	MDM4	4.00%	3	**CTNNB1**	1.33%	1
FGF19	30.67%	23	MLL2	4.00%	3	**EGFR**	1.33%	1
FGF3	30.67%	23	RAD50	4.00%	3	ETV6	1.33%	1
**FGF4**	30.67%	23	**RB1**	4.00%	3	FANCA	1.33%	1
**ESR1**	26.67%	20	RUNX1	4.00%	3	**FGFR4**	1.33%	1
MYC	24.00%	18	**ATR**	2.67%	2	**FLT3**	1.33%	1
CDH1	18.67%	14	CCND2	2.67%	2	**FLT4**	1.33%	1
**PTEN**	18.67%	14	CREBBP	2.67%	2	**HRAS**	1.33%	1
EMSY	14.67%	11	EPHB1	2.67%	2	**JAK2**	1.33%	1
ZNF217	14.67%	11	FGF14	2.67%	2	KDM5A	1.33%	1
GATA3	12.00%	9	FGF23	2.67%	2	**MAP2K2**	1.33%	1
MYST3	12.00%	9	FGF6	2.67%	2	MEN1	1.33%	1
**FGFR1**	10.67%	8	IKBKE	2.67%	2	MITF	1.33%	1
ARFRP1	9.33%	7	**JUN**	2.67%	2	MSH2	1.33%	1
**MAP2K4**	9.33%	7	**KDR**	2.67%	2	MYCL1	1.33%	1
**TOP1**	9.33%	7	**KIT**	2.67%	2	MYCN	1.33%	1
CCNE1	8.00%	6	MCL1	2.67%	2	**NF1**	1.33%	1
**FGFR2**	8.00%	6	NOTCH1	2.67%	2	NFKBIA	1.33%	1
MAP3K1	8.00%	6	**PDGFRB**	2.67%	2	NKX2-1	1.33%	1
AURKA	6.67%	5	**RPTOR**	2.67%	2	PALB2	1.33%	1
CDKN2A	6.67%	5	SPEN	2.67%	2	PAX5	1.33%	1
CDKN2B	6.67%	5	**STK11**	2.67%	2	**PIK3R1**	1.33%	1
**ERBB2**	6.67%	5	ZNF703	2.67%	2	PRKDC	1.33%	1
MDM2	6.67%	5	**AKT1**	1.33%	1	**RARA**	1.33%	1
ARID1A	5.33%	4	**AKT2**	1.33%	1	SMAD4	1.33%	1
ATM	5.33%	4	**AKT3**	1.33%	1	SMARCA4	1.33%	1
CCND3	5.33%	4	APC	1.33%	1	**SRC**	1.33%	1
**GNAS**	5.33%	4	ARID2	1.33%	1	SUFU	1.33%	1
**IGF1R**	5.33%	4	ATRX	1.33%	1	**TET2**	1.33%	1
**KRAS**	5.33%	4	BCL2L2	1.33%	1	**TSC2**	1.33%	1
SOX2	5.33%	4	**BRAF**	1.33%	1	**VHL**	1.33%	1
BRCA2	4.00%	3	BRIP1	1.33%	1			
CDK12	4.00%	3	**BTK**	1.33%	1			

Note: Genes in **bold** have at least 1 FDA approved drug recommendation. Genes with underline have at least 1 clinical trial recommendation from multiple sources.

**Figure 1:**
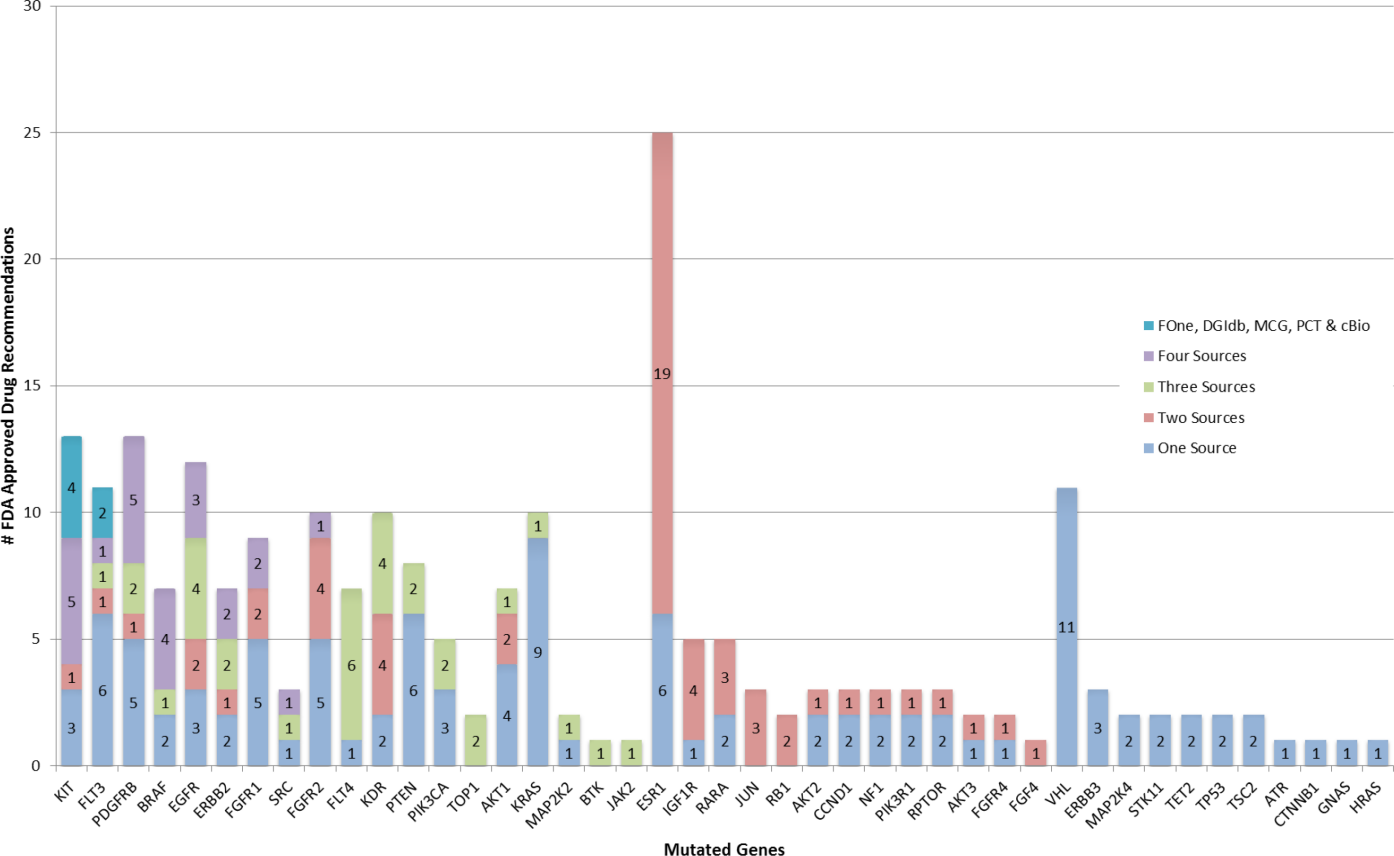
Occurrence of FDA approved drug recommendations Mutated genes from highest to lowest number of sources are listed along x-axis and number of drug recommendations grouped by number of contributing sources are stacked along y-axis.

However, for a given mutated gene, a drug recommendation by one source did not imply identical recommendation from another source. Only for 2 genes (KIT, FLT3) did all 5 sources recommended the same drug. Three other genes (BRAF, FGFR1, FGFR2,) had drug recommendation from all 5 sources, but the recommendations were partially discordant (Figure 1 and Table 2). There were 7 genes (PDGFRB, BRAF, EGFR, ERBB2, FGFR1, SRC & FGFR2) with the same drug recommendation from 4 sources and 11 genes with drug recommendations from 3 sources; among these 10 had at least one drug recommended by all 3 sources. There were 12 genes for which only 2 web sites made drug recommendations, but 11 of these genes had the same drug recommended by both. These results indicate only partial overlap in identifying similar treatment options based on the same mutation data among different web-based tools and FoundationOne.

**Table 2:** FDA approved drug recommendations listed by mutation and source

Gene	Foundation	DGIdb	MCG	PCT	cBioPortal
**AKT1**	Everolimus, Temsirolimus	Everolimus, Temsirolimus, Nelfinavir, Risperidone		Everolimus, Temsirolimus, Metformin, Sirolimus	Arsenic Trioxide
**AKT2**	Everolimus, Temsirolimus	Everolimus			
**AKT3**	Everolimus, Temsirolimus	Everolimus			
**ATR**		Olaparib			
**BRAF**	Sorafenib, Trametinib, Regorafenib	Sorafenib, Dabrafenib, Vemurafenib, Regorafenib, Trametinib, Dasatinib, Temsirolimus	Dabrafenib, Vemurafenib, Trametinib	Sorafenib, Dabrafenib, Vemurafenib, Regorafenib, Trametinib	Sorafenib, Dabrafenib, Vemurafenib
**BTK**		Ibrutinib	Ibrutinib		Ibrutinib
**CCND1**		Arsenic Trioxide, Methotrexate			Arsenic Trioxide
**CTNNB1**		Celecoxib			
**EGFR**	Afatinib, Erlotinib, Gefitinib, Cetuximab, Lapatinib, Panitumumab	Afatinib, Erlotinib, Gefitinib, Cetuximab, Lapatinib, Panitumumab, Vandetanib, Lidocaine, Trastuzumab, Bevacizumab, Pazopanib, Pertuzumab	Afatinib, Erlotinib, Gefitinib, Vandetanib		Afatinib, Erlotinib, Gefitinib, Cetuximab, Lapatinib, Panitumumab, Vandetanib, Lidocaine, Trastuzumab
**ERBB2**	Afatinib, Lapatinib, Pertuzumab, Trastuzumab, Ado-trastuzumab	Afatinib, Lapatinib, Pertuzumab, Trastuzumab, Ado-Trastuzumab, Bevacizumab, Gefitinib	Afatinib, Lapatinib		Afatinib, Lapatinib, Pertuzumab, Trastuzumab
**ERBB3**	Afatinib, Pertuzumab	Gefitinib			
**ESR1**		Clomiphene, Danazol, Desogestrel, Estramustine, Estropipate, Ethinyl Estradiol, Ethynodiol Diacetate, Fluoxymesterone, Fulvestrant, Levonorgestrel, Medroxyprogesterone, Megestrol, Naloxone, Norgestimate, Norgestrel, Progesterone, Raloxifene, Tamoxifen, Toremifene, Anastrozole, Cisplatin, Exemestane, Leflunomide, Letrozole			Clomiphene, Danazol, Desogestrel, Estramustine, Estropipate, Ethinyl Estradiol Ethynodiol Diacetate, Fluoxymesterone, Fulvestrant, Levonorgestrel, Medroxyprogesterone, Megestrol, Naloxone, Norgestimate, Norgestrel, Progesterone, Raloxifene, Tamoxifen, Toremifene, Bazedoxifene
**FGF4**		Pentosan Polysulfate			Pentosan Polysulfate
**FGFR1**	Regorafenib, Ponatinib, Pazopanib	Regorafenib, Ponatinib, Palifermin	Ponatinib, Nintedanib	Regorafenib, Ponatinib, Pazopanib, Dasatinib, Sorafenib, Sunitinib, Vandetanib	Regorafenib, Palifermin
**FGFR2**	Ponatinib, Pazopanib	Ponatinib, Palifermin, Regorafenib, Thalidomide, Interferon, Pentosan Polysulfate	Ponatinib, Nintedanib	Ponatinib, Pazopanib, Regorafenib, Sorafenib, Sunitinib	Palifermin, Thalidomide
**FGFR4**	Ponatinib	Palifermin			Palifermin
**FLT3**	Sorafenib, Sunitinib, Ponatinib	Sorafenib, Sunitinib, Ponatinib, Cabozantinib, Azacitidine, Bortezomib, Clofarabine, Idarubicin	Sorafenib, Sunitinib, Ponatinib, Cabozantinib	Sorafenib, Sunitinib, Ponatinib, Cabozantinib, Vandetanib, Pazopanib, Regorafenib	Sorafenib, Sunitinib, Vandetanib
**FLT4**	Axitinib, Pazopanib, Regorafenib, Sorafenib, Sunitinib, Vandetanib	Axitinib, Pazopanib, Regorafenib, Sorafenib, Sunitinib, Vandetanib			Axitinib, Pazopanib, Regorafenib, Sorafenib, Sunitinib, Vandetanib, Cabozantinib
**GNAS**	Trametinib				
**HRAS**	Trametinib				
**IGF1R**		Glargine Insulin, Lispro Insulin, Porcine Insulin			Glargine Insulin, Lispro Insulin, Porcine Insulin, Mecasermin
**JAK2**		Ruxolitinib	Ruxolitinib		Ruxolitinib
**JUN**		Arsenic Trioxide, Irbesartan, Vinblastine			Arsenic Trioxide, Irbesartan, Vinblastine
**KDR**	Pazopanib, Sorafenib, Sunitinib, Vandetanib, Axitinib, Regorafenib, Bevacizumab	Pazopanib, Sorafenib, Sunitinib, Vandetanib, Cabozantinib, Ramucirumab, Regorafenib, Ponatinib			Pazopanib, Sorafenib, Sunitinib, Vandetanib, Axitinib, Cabozantinib, Ramucirumab
**KIT**	Dasatinib, Imatinib, Pazopanib, Regorafenib, Sunitinib, Nilotinib, Sorafenib, Ponatinib, Everolimus, Temsirolimus	Dasatinib, Imatinib, Pazopanib, Regorafenib, Sunitinib, Axitinib, Nilotinib, Sorafenib, Cabozantinib	Dasatinib, Imatinib, Pazopanib, Regorafenib, Sunitinib, Axitinib, Cabozantinib	Dasatinib, Imatinib, Pazopanib, Regorafenib, Sunitinib, Axitinib, Nilotinib, Sorafenib, Cabozantinib, Pazopanib, Ponatinib	Dasatinib, Imatinib, Pazopanib, Regorafenib, Sunitinib, Axitinib, Nilotinib, Sorafenib, Cabozantinib, Vandetanib
**KRAS**	Trametinib	Trametinib, Cetuximab, Everolimus, Erlotinib, Gefitinib, Panitumumab, Regorafenib, Simvastatin, Temsirolimus, Vandetanib		Trametinib	
**MAP2K2**	Trametinib	Trametinib, Dabrafenib			Trametinib
**MAP2K4**		Trametinib, Dabrafenib			
**NF1**	Everolimus, Temsirolimus, Trametinib	Trametinib			
**PDGFRB**	Imatinib, Pazopanib, Regorafenib, Sorafenib, Sunitinib, Dasatinib, Everolimus, Ponatinib, Temsirolimus	Imatinib, Pazopanib, Regorafenib, Sorafenib, Sunitinib, Axitinib, Dasatinib, Nilotinib	Imatinib, Pazopanib, Regorafenib, Sorafenib, Sunitinib, Axitinib, Nintedanib		Imatinib, Pazopanib, Regorafenib, Sorafenib, Sunitinib, Axitinib, Dasatinib, Nilotinib, Cabozantinib
**PIK3CA**	Everolimus, Temsirolimus	Everolimus, Temsirolimus, Docetaxel		Everolimus, Temsirolimus, Sirolimus, Metformin	
**PIK3R1**	Everolimus, Temsirolimus	Isoproterenol			Isoproterenol
**PTEN**	Everolimus, Temsirolimus	Everolimus, Temsirolimus, Cetuximab, Erlotinib, Gefitinib, Panitumumab, Vandetanib		Everolimus, Temsirolimus, Sirolimus	
**RARA**		Acitretin, Etretinate, Isotretinoin, Arsenic Trioxide, Tretinoin			Acitretin, Etretinate, Isotretinoin
**RB1**		Porcine Insulin, Recombinant Insulin			Porcine Insulin, Recombinant Insulin
**RPTOR**	Everolimus, Temsirolimus	Sirolimus, Temsirolimus			
**SRC**	Dasatinib, Bosutinib	Dasatinib, Bosutinib, Ponatinib	Dasatinib		Dasatinib, Bosutinib
**STK11**	Everolimus, Temsirolimus				
**TET2**	Azacitidine, Decitabine				
**TOP1**	Irinotecan, Topotecan	Irinotecan, Topotecan			Irinotecan, Topotecan
**TP53**		Fluorouracil, Paclitaxel			
**TSC2**	Everolimus, Temsirolimus				
VHL	Axitinib, Cabozantinib, Everolimus, Pazopanib, Ponatinib, Ramucirumab, Regorafenib, Sorafenib, Temsirolimus, Sunitinib, Vandetanib				

Note: Drugs with overlap in recommendation sources are listed in bold for four-way overlap and in bold + underline for five-way overlap.

**Figure 2:**
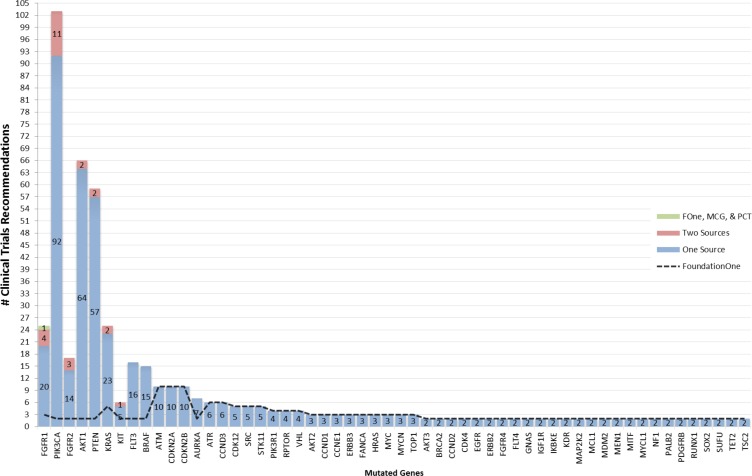
Occurrence of clinical trial recommendations Mutated genes from highest to lowest number of sources are listed along x-axis and number of clinical trials grouped by number of contributing sources are stacked along y-axis.

The median numbers of drugs for a targetable gene identified by the different tools was 2 for Foundation One (range 1–11), 3 for DGIdb (range: 1–24), 5 for PCT (range: 1–10), 2 for MCG (range 1–7) and 2 for cBioPortal (range: 1–20). Clinical trial recommendations were reported for 56 of the 103 genes by FoundationOne. Only 7 of these genes (AKT1, BRAF, FGFR1, FGFR2, KIT, PIK3CA & PTEN) had at least one clinical trial that was recommended by both the FoundationOne report and another source (Figure 2). The median number of clinical trials recommended by FoundationOne (for 56 genes), MCG (for 5 genes) and PCT (for 9 genes) was 2, 51 and 9 respectively.

**Figure 3:**
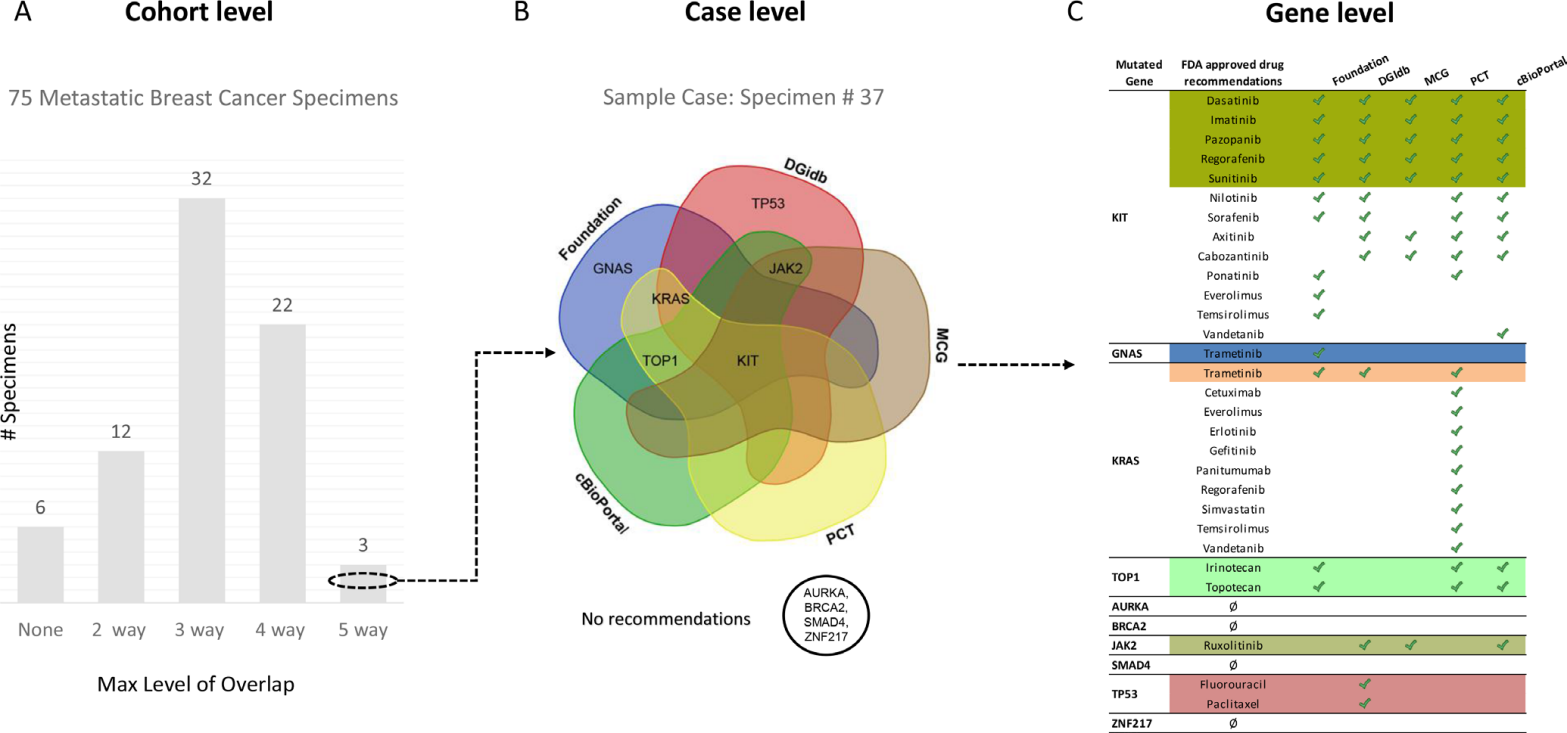
Overview of overlapping FDA approved drug recommendations at multiple levels (**A**) 75 metastatic breast cancer specimens grouped by maximum level of overlapping FDA approved drug recommendation(s) for any of its mutated genes. (**B**) Venn diagram showing relationship between four recommendation sources (FoundationOne, Drug Gene Interaction Database, Personalized Cancer Therapy and cBioPortal) with mutated genes in sample specimen # 37 [KIT, GNAS, KRAS, TOP1, AURKA, BRCA2, JAK2, SMAD4, TP53, ZNF217] based on maximum overlapping FDA approved drug recommendation(s). (**C**) Table listing all mutated genes for sample specimen # 37 (column 1), their FDA approved drug recommendations (column 2), sources identified by green check mark (column 3–6) and a colored background that corresponds with maximum overlapping drug(s) represented in (B).

For individual cases, the drug or clinical trial recommendations ranged from high degree of overlap to no overlap across the 5 tools. Figure 3 illustrates recommendations at the cohort level, case level and gene level for a single case. This cancer had 10 mutated genes with multiple drug recommendations that illustrate the various degrees of overlap in treatment options. For example, KIT had identical recommendations by all 5 tools, KRAS, TOP1 and JAK2 had 3 tools reporting the same drug as an option, while GNAS and TP53 each had drug recommendations by FoundationOne only. Alterations in AURKA, BRCA2, SMAD4 & ZNF217 had no drug recommendations. Overall, 3 cases had at least 1 gene with an identical drug recommendation from all 5 sources, 22 cases with an overlapping recommendation from 4 sources, 32 cases from 3 sources, 12 cases from 2 sources and 6 with no overlapping recommendations at all. Additionally, 21 cases had at least 1 overlapping clinical trial recommendation from 3 sources, 33 cases had 2 sources and 21 cases had at least 1 gene with a clinical trial recommendation from FoundationOne, but no recommendations from any of the other sources. In summary, 33% of the metastatic breast cancer cases had a drug recommendation for at least 1 of its mutated genes that was agreed upon by 4 or more of the sources and 72% had a clinical trial recommendation agreed upon by 2 or more of the sources.

## DISCUSSION

The purpose of this study was to examine to what extent different web tools and a widely used commercial service identify similar therapeutic options for a given set of genomic anomalies in a cancer. Our data represents the typical clinical scenario of tumor only research biopsies obtained mostly from a metastatic site in heavily pretreated patients. Target profiling was performed by FoundationOne assay. We observed only partial overlap in finding similar treatment options based on the same mutation data among 5 recommendation sources designed to enable personalized medicine. Only 33% of cases had a drug recommendation agreed upon by 4 or more sources for at least 1 mutated gene. The different tools identified different number of genes as targetable with off-label use of FDA-approved drugs. Using the same input data from the FoundationOne reports, DGIdb identified 36 genes, FoundationOne identified 31 genes, cBioPortal 25 genes, MCG 10 genes and PCT 9 genes as potentially targetable. Furthermore, clinical trial recommendations were provided by only half of the web tools (MCG & PCT) and were less frequently provided for both web tools combined (14 genes) than FoundationOne alone (56 genes). While we recognize that identical treatment recommendations for a given molecular abnormality from multiple sources does not imply clinical validity or increase the likelihood that the suggested treatment would work, our findings highlight the challenges in interpreting clinical tumor profiling results.

Different therapeutic conclusions can be drawn from the same data depending on what analytic tool is used. This is primarily due to the different rules used by different tools to define what constitutes a druggable gene. Concordance was greater when there was high level of evidence from clinical trials that a given drug directly targets a particular molecular abnormality (e.g. Kit, BRAF, ERBB2). However, for the majority of mutations and affected genes no such drug exists. In these instances, potential drug efficacy is inferred from biological principles or from off-target effects of kinase inhibitors. Different websites apply different rules to such circumstantial evidence that largely explains the substantial discordance. For example, the most frequently, but not the most concordantly recommended drug, was the mTOR inhibitor temsirolimus for alterations in PIK3CA, PIK3R1, PTEN, STK11, AKT-1, -2-, 3, TSC2, KIT, PDGFRB, KRAS, VHL, RPTOR, NF1 and BRAF based on biological pathway-level association. The effect of temsirolimus on these targets is hypothetical since no mutations in any of these genes have been directly linked to temsirolimus sensitivity in patients, therefore discordant recommendations are not unexpected. A more concerning example is FGFR4 amplification. One source identified ponatinib, and two other tools identified palifermin (but not ponatinib) as potential options, among other drugs. Neither of these drugs represents a valid option. Ponatinib is a tyrosine kinase inhibitor that primarily targets BCR-ABL but also inhibits the FGFR receptor family; however, it has been withdrawn from the market due to serious toxicity concerns in 2013 [19]. Palifermin is a truncated recombinant keratinocyte growth factor (KGF), which mimics the actions of endogenous KGF by binding to and activating the fibroblast growth factor receptor 2b (FGFR2b) [20]. There is no plausible biological hypothesis, or preclinical, data suggesting that palifermin would work as an anticancer drug for FGFR4-amplified cancers.

Since drug approval status and clinical trial options evolve rapidly as does our understanding of molecular pathways and drug targets, expert curation is currently still critical for the accuracy of the data. Unfortunately, human review of data elements is low throughput, time consuming and may also be of variable quality. The annotation databases also often lack information on the effect of gene level alterations (e.g. loss-of-function mutation vs amplification or gain-of-function mutations) on the biological pathway activity that is involved. A further difficulty arises from the presence of multiple mutations in different genes in the same cancer which may result in multiple different drug recommendations (Figure 3). This is consistent with the notion that ultimately combinations of targeted drugs will be required for successful therapy [21]. However, most of the drug combinations that would match the molecular abnormalities in a particular cancer have never been tested formally for safety in a Phase I trial.

In summary, we examined 4 different websites and a commercial service that were designed to link mutated genes to potential therapeutic options and found only partial overlap in the treatment options that were identified for the same genomic abnormalities. The highly curated sites contain more accurate information but only cover a very small number of genes, while the sites that are more broadly applicable are also more error prone and can include outdated information or link mutations to drugs that are not supported by strong evidence. Further improvements in search algorithms, data integration from multiple sources, and rapid and real-time interpretation of the peer-reviewed literature combined with artificial intelligence tools will be required for the development of broadly applicable software tools to assist therapeutic interpretation of high throughput genomic data [22, 23].

## MATERIALS AND METHODS

### Patients and molecular data

Molecular target profiling was performed in the context of a clinical study (clinicaltrial.gov: NCT01855503) to identify potentially actionable molecular abnormalities in prospectively collected metastatic tumor biopsy specimens of breast cancer (*n* = 33 liver, *n* = 13 lymph node, *n* = 5 breast, *n* = 5 skin, *n* = 5 soft tissue, *n* = 3 lung, *n* = 3 ovary, *n* = 8 other). The study was approved by the Human Research Committee of the Yale Cancer Center. Seventy-five patients were accrued between June 2013 and June 2015. Target profiling was performed using the FoundationOne^™^ (Foundation Medicine Inc. Cambridge, MA) targeted sequencing assay that interrogates the coding sequence of 315 cancer-related genes and select introns from 28 genes often rearranged in solid tumors [1, 4]. The assay report includes only molecular abnormalities that are deemed potentially actionable using a proprietary method and lists the drugs and clinical trials that represent therapeutic options (Supplementary Table S1 lists the specific abnormalities for each cases and the location of the biopsy).

### Web tools

We ran the results from each case through 4 different websites that were designed to link mutated genes to potential therapeutic options. Table 3 presents an overview of the websites. The *Drug-Gene Interaction Database* (DGIdb) is affiliated with Washington University School of Medicine (http://dgidb.genome.wustl.edu) and integrates data from 13 primary sources to provide over 14,144 drug-gene interactions involving 2,611 genes and 6,307 drugs [13]. It's intended for researchers and has a specific disclaimer that the information is not to be used for medical advice. The input information is any gene symbol and the output is a list of generic, brand or developmental code names of drugs that are unfiltered for duplicates. The result also includes the predicted effect of the chemical entity on gene function (i.e. activator or inhibitor) and the name of the source database. This website does not identify clinical trials options. The *My Cancer Genome* (MCG) website is affiliated with Vanderbilt-Ingram Cancer Center (http://www.mycancergenome.org/) and provides extensive background information and potential clinical trial options for specific mutations in 55 genes in 21 different cancer types [14]. It is based on manual curation by physician-scientists and is intended to provide clinically relevant information for patients, and clinical researchers. During the query, the cancer type and gene is selected from a dropdown menu and clinical trial options are listed through the clinicaltrials.gov website using the NCT identifier number and study title. Specific drug recommendations are also provided since 2015. The *Personalized Cancer Therapy* (PCT) website is affiliated with MD Anderson Cancer Center (https://pct.mdanderson.org/). It includes detailed biological information in various disease contexts on 20 genes that can be selected from a dropdown menu. It relies both on manual curation and automatic database mining [15]. The intended audience is both patients and clinical researchers. Only clinically accessible drugs (approved or in active clinical trials) are listed and links to clinical trials are provided through the clinicaltrials.gov website. The *cBioPortal* (http://www.cbioportal.org/public-portal/) is affiliated with Memorial Sloan Kettering Cancer Center and provides access to a variety of information on 17,584 tumor samples from 69 cancer studies [16–18]. It integrates data from a large number of diverse sources and is intended audience are researchers. For a query, cancer data type and assay platform must be selected and a gene symbol entered. The output is presented as interactive molecular and epidemiologic data drawn from the selected database and data platform and can be accessed through various tabs which lead to tables and graphical results. Drugs that interact with the selected gene (either in preclinical or clinical experiments) can be found under the “network” tab and are embedded in an interactive gene network diagram; drug information can be filtered by FDA approval status. There is no listing of clinical trials. For this study, a gene was queried selecting the Breast cancer - TCGA (provisional) database and mutation and copy number level alterations, and potential drugs were identified through the network function.

**Table 3:** Comparison of 5 mutation based treatment recommendation sources

Source	FoundationOne^®^ www.foundationone.com	My Cancer Genome^™^ www.mycancergenome.org	Personalized Cancer Therapy pct.mdanderson.org	Drug Gene Interaction Database www.dgidb.org	cBioPortal www.cbioportal.org
**Affiliation**	commercial	Vanderbilt-Ingram Cancer Center	MD Anderson Cancer Center	Washington University School of Medicine in St. Louis	Memorial Sloan Kettering Cancer Center
**Access**	proprietary	free website	free website	free website	free website
**Intended Audience**	patients clinicians	patients clinicians researchers	patients clinicians	researchers only	researchers only
**Genes Covered**	315	55^*^	16	> 2000	> 2000
**Data Entry**	n/a	dropdown list^*^	dropdown list	search box	search box
**Recommendations**	FDA approved drugs clinical trials	clinical trials	FDA approved drugs investigational drugs clinical trials	FDA approved drugs investigational drugs chemical compounds	FDA approved drugs investigational drugs chemical compounds
**Output**	Drugs according to mutated gene with particular variant are listed in a table by generic name based on FDA approval for patient's malignancy versus any other malignancies.	Drugs according to mutated gene are listed in a table by generic, trade, code and/or chemical name.	Drugs according to mutated gene are listed in a table by generic, code or chemical name along with columns for alternative names, molecular targets, FDA indications if applicable and phase of development.	Drugs according to mutated gene are listed in a table unfiltered for duplicates & unlabeled by FDA status by generic, brand, code or chemical name along with predicted interaction and data source.	Drugs according to mutated gene are each uniquely distributed on an interactive gene network diagram by a connecter line that indicates predicted level of action on network and FDA status based on color of connecter.
	Clinical trials according to mutated gene with particular variant are listed in a table with study title, trial phase, available locations and NCT identifier number.	Clinical trials according to mutated gene in specific malignancy are listed in a tabular format subdivided by location (US, International, Unknown) with NCT identifier number study title & trial phase.	Clinical trials according to mutated gene are listed in a table by NCT identifier number and study title along with a link for more information.		
**Algorithm**	proprietary	Internal expert ­curation of multiple databases.	Combination of internal natural language processing code and internal expert curation of multiple databases.	Combination of open source code & internal expert curation of both externally curated and uncurated databases.	Combination of open source code and internal expert curation of externally curated databases and 69 cancer datasets.
**Background Source**	brief description & primary reference for drug provided	background information and primary reference on mutated gene provided	brief description & primary reference for drug provided	name of drug database provided	name of drug database provided
**Last update noted**	n/a	Yes	Yes	Yes	Yes

* Total number of genes listed under any 1 of 21 malignancies, excluding repeats. Note: Gene list & access to information is restricted by required selection of malignancy.

### Data collection and analysis plan

Treatment options were retrieved from all 4 web sites for all cases by entering every altered gene that was detected in a given cancer and reported by Foundation One. Abnormalities were collapsed at gene level because none of the sites were designed to interpret specific variants (with a few exception of canonical oncogenic mutations). Results were categorized as either FDA approved drugs (http://www.accessdata.fda.gov/scripts/cder/drugsatfda/index.cfm) or clinical trial options defined by the National Clinical Trial (NCT) identifier. We use the term FDA-approved drug to indicate commercial availability under some cancer indication; we do not imply that a given drug is approved for breast cancer. We did not consider drugs or molecules that are not commercially available. Duplicate entries and drug name aliases were removed to create a single entry for each drug or trial. We assigned drug targets to “primary” or “secondary” categories whether the target represented the presumed main mechanism of action of a drug or an ancillary effect. The biopsies were collected over a 24 months period and the drugs and clinical trial options reported in the results reflect the options that were available when the test was performed. To adjust for possible time-related discordance in treatment options, we reviewed each case for this possible bias. Websites were last accessed on August 7, 2015.

## SUPPLEMENTARY FIGURES AND TABLES


