# Supplementary material for: Mutation based treatment recommendations from next generation sequencing data: a comparison of web tools
Source: Oncotarget. 2016 Mar 9;7(16):22064–76. doi: 10.18632/oncotarget.8017 (PMC5008344; doi:10.18632/oncotarget.8017)
Supplement: Supplementary file 1 [file oncotarget-07-22064-s001.pdf]

Mutation based treatment recommendations from next generation sequencing data: a comparison of web tools

Supplementary Materials

Supplementary Table S1: Lists the specific abnormalities for each cases and the location of the biopsy

Specimen #	Biopsy Site	Gene	Variant
1	lymph node	PTEN	loss exons 2-9
		TET2	T1114fs*16
		TP53	R209fs*6
		CDH1	K268fs*1
		RAD50	S181fs*7
2	liver	ERBB2	V777L, V842I
		ERBB3	E928G
		CDH1	T522fs*35
3	skin	PIK3R1	A331fs*2
		STK11	loss exon 1
		CCNE1	amplification
		TP53	R110P
4	liver	KRAS	amplification
		CCND2	amplification
		MDM2	amplification
		MYC	amplification
		TP53	R273H
		MAP2K4	loss
		FGF6	amplification
		FGF23	amplification
		KDM5A	amplification
5	lymph node	PTEN	truncation exon 1
		CCNE1	amplification
		TP53	E294*
6	liver	PIK3CA	H1047R
		FGFR1	amplification
		TOP1	amplification
		CDKN2A	loss
		CDKN2B	loss
		ARFRP1	amplification
		ZNF703	amplification
		ZNF217	amplification
7	liver	PIK3CA	E542K
		CREBBP	Q513*

		SPEN	S659*
8	liver	PTEN	T319fs*1
		TP53	H179Y
		EPHB1	amplification
		ESR1	Y537C
		ARFRP1	amplification
		LRP1B	splice site 3521-1G>T
9	lymph node	TP53	K120E
		Notch1	Y967*
		CDH1	loss
10	liver	PIK3CA	H1047R
		PTEN	T319fs*1
		MYCN	amplification
		SOX2	amplification
		CDKN2A	loss
		CDKN2B	loss
		TP53	R175H
		CCND1	amplification
		APC	truncation exon 9
		FGF19	amplification
		FGF4	amplification
		FGF3	amplification
		CARD11	S652L
11	head and neck	KRAS	amplification
		FLT3	amplification
		MDM2	amplification
		CCND1	amplification
		CCND2	amplification
		TP53	M246L
		FGF19	amplification
		FGF23	amplification
		FGF3	amplification
		FGF4	amplification
		FGF6	amplification
		GATA3	G335fs*18
12	liver	TOP1	amplification
		CCND1	amplification
		MYC	amplification
		ESR1	Y537S
		ARID1A	S264*
		FGF19	amplification
		FGF4	amplification
		FGF3	amplification
		RAD50	L424fs*7
13	ovary	PTEN	N311fs*4
		TP53	H179R
		EMSY	amplification

		MAP2K4	loss
14	unknown	ERBB2	amplification
		PIK3CA	H1047R
		MAP2K2	amplification
		MYC	amplification
		TP53	L348*
		FGF14	amplification
15	liver	ERBB2	D769Y
		PIK3CA	E453_D454del
		ESR1	L536_Y537insN
		MAP3K1	T829fs*10
16	liver	MYC	amplification
		TP53	slice site 783-1G>T
		RB1	complex rearrangement
		CREBBP	truncation
17	liver	PIK3CA	E545K
		CCND1	amplification
		ESR1	Y537N
		MDM4	amplification
		FGF4	amplification
		FGF19	amplification
		FGF3	amplification
18	lymph node	EGFR	amplification
		CCND3	amplification
		TP53	R248W
19	liver	RUNX1	L225fs*12
		ARID1A	T2105fs*35
		BTK	R492H
		ESR1	Y537S
		ATRX	splice
		MAP3K1	R273fs*27, Q535fs*5
20	soft tissue	PIK3CA	D1017H, G1049R
		CDK4	amplification
		TP53	S241fs*
		CDH1	V458fs*5
21	liver	PIK3CA	amplification, E545K
		FGFR1	amplification
		TP53	P27fs*17
		ZNF703	amplification
22	lymph node	CCND3	amplification
		MYC	amplification
		TP53	Y220C
		BCL2L2	amplification
		JUN	amplification
		MYST3	amplification
23	chest wall	PIK3CA	H1047R
		ATM	splice site 2921+1G>A

		MAP3K1	F1149fs*25, R1278fs*11
24<br>25	skin	AKT1	amplification, E17K
		TP53	Q136*
	chest wall	TSC2	T849fs*24
		TP53	R306*
		RARA	RARA-LOC400931 fusion
26	breast	PIK3CA	amplification
		KIT	amplification
		KDR	amplification
		SOX2	amplification
		IGF1R	amplification
		CCND1	amplification
		ATM	E71*
		TP53	deletion exons 10-11, R156C
		EPHB1	amplification
		ESR1	amplification
		MAP2K4	W291*
		GATA3	S438fs*9+
		LRP1B	R2066fs*54
		FGF19	amplification
		FGF4	amplification
		FGF3	amplification
		ZNF217	amplification
		MYST3	amplification
27	liver	TP53	R248Q
		RAD50	E1030_L1031>D*EL
28	breast	PIK3CA	H1047R
		PTEN	C71Y, loss
		ERBB3	E928G
		FGFR2	amplification
		BRCA2	K3326*
		SUFU	deletion exons 9-11
		CCND1	amplification
		FGF3	amplification
		FGF4	amplification
		FGF19	amplification
29	liver	GNAS	amplification
		IGF1R	amplification
		MYC	amplification
		ESR1	Y537N
		ARFRP1	amplification
		GATA3	S405fs*42+
		ZNF217	amplification
30	soft tissue	PIK3CA	E545K
		TP53	C141_V143del
		CDKN2A	loss
		CDKN2B	loss

		MCL1	amplification
		SMARCA4	splice site 1119-1_1119insAGCCTGCGCAG
		SPEN	E103fs*27
31	liver	PTEN	R130G
		TOP1	amplification
		ESR1	D538G
		MAP2K4	loss
		GATA3	I407fs*36+
		ARFRP1	amplification
		ZNF217	amplification
32	liver	FGFR2	truncation exon 17
		MYC	amplification
		MDM2	amplification
		BRCA2	V1681fs*7
		MAP3K1	loss
33	breast	FGFR1	amplification
		CCND1	amplification
		MYC	amplification
		EMSY	amplification
		FGF19	amplification
		FGF4	amplification
		FGF3	amplification
		ZNF703	amplification
34	lymph node	ERBB2	amplification
		CDK12	rearrangement, exon 14
		MDM4	amplification
		PAX5	TPM2-PAX5 fusion
		ZNF217	amplification
35	liver	PIK3CA	H1047R
		ATM	E3022fs*37+
		ESR1	Y537S
36	ovary	PIK3CA	E545K
		CDH1	I650fs*13
37	peritoneum	KIT	amplification
		GNAS	amplification
		KRAS	amplification
		TOP1	amplification
		AURKA	amplification
		BRCA2	T3033fs*29
		JAK2	E1097*
		SMAD4	loss exons 2-4
		TP53	loss exons 8-9
		ZNF217	amplification
38	liver	PIK3CA	E545K
		CCND1	amplification
		MYC	amplification
		TP53	R248G

		EMSY	amplification
		FGF19	amplification
		FGF3	amplification
		FGF4	amplification
39	liver	PIK3CA	H1047R
		CCND1	amplification
		CDH1	D400Y
		EMSY	amplification
		FGF19	amplification
		FGF3	amplification
		FGF4	amplification
		LRP1B	R590fs*4
40	lymph node	FGFR2	K659E
		CCND1	amplification
		FGF19	amplification
		FGF3	amplification
		FGF4	amplification
41	liver	STK11	loss
		CCNE1	amplification
		MYC	amplification
		TP53	H297fs*48
42	lung	PTEN	N323fs*2
		FGFR2	amplification
		TOP1	amplification
		ARFRP1	amplification
		MAP2K4	S240fs*29
		ZNF217	amplification
43	lymph node	AKT3	amplification
		PDGFRB	amplification
		PIK3CA	H1047R
		FGFR4	amplification
		FLT4	amplification
		TOP1	amplification
		IKBKE	amplification
		MDM2	amplification
		MDM4	amplification
		ZNF217	amplification
44	liver	PIK3CA	C407W
		TP53	F109_R110del
		FGF14	amplification
		PRKDC	R1136H
45	liver	AKT2	amplification
		CCNE1	amplification
		TP53	R175H
		CTNNB1	A636fs*12
		MYST3	amplification
46	liver	PIK3CA	amplification, H1047R

		HRAS	Q61K
		KRAS	Q61K
		TP53	G245D
47	liver	FGFR2	S252W
		GNAS	amplification
		PIK3CA	H1047R
		AURKA	amplification
		CCND1	amplification
		TP53	K132N
		FGF19	amplification
		FGF3	amplification
		FGF4	amplification
48	lung	PIK3CA	amplification
		CCND3	amplification
		IKBKE	amplification
		TP53	W91*
		SOX2	amplification
49	breast	CCND1	amplification
		EMSY	amplification
		FGF19	amplification
		FGF3	amplification
		FGF4	amplification
50	lymph node	RPTOR	amplification
		FGFR1	amplification
		CCND1	amplification
		MYC	amplification
		EMSY	amplification
		FGF19	amplification
		FGF3	amplification
		FGF4	amplification
		MYST3	amplification
		ZNF703	amplification
51	liver	PTEN	Loss exons 1,2
		FANCA	rearrangement, intron 7
		MCL1	amplification
		TP53	C242fs*5
52	head and neck	PIK3CA	E545Q, H1047R
		RUNX1	R201Q
		MAP3K1	W560*
53	soft tissue	CCND1	amplification
		ESR1	Y537C
		FGF19	amplification
		FGF3	amplification
		FGF4	amplification
54	liver	ERBB2	amplification
		ERBB3	amplification
		PTEN	Q171*

		VHL	F76L
		CCND3	amplification
		CDK12	truncation exon 2
		MYC	amplification
		TP53	R65fs*58
		MLL2	E551*
		RB1	loss exons 7-17
55	lung	ATR	F926fs*13
		CCNE1	amplification
		TP53	H179R
		ESR1	D538G
		MAP2K4	loss
		RB1	splice site 2107-1G>T
56	liver	PDGFRA	P567L
		IGF1R	amplification
		MYC	amplification
		TP53	Y126*
		MAP2K4	R287H
		MYST3	amplification
57	liver	CCND1	amplification
		FGFR1	amplification
		EMSY	amplification
		ESR1	D538G
		FGF19	amplification
		FGF3	amplification
		FGF4	amplification
		MYST3	amplification
		ZNF703	amplification
58	liver	PIK3CA	Q546R
		CCND1	amplification
		FGF19	amplification
		FGF3	amplification
		FGF4	amplification
59	lymph node	TP53	G108fs*15
		ETV6	truncation intron 5 & dupl. introns 6-8
60	abdomen	PTEN	C83fs*18
		CCND1	amplification
		FGFR1	amplification
		MYC	amplification
		CDH1	D587fs*2
		EMSY	amplification
		ESR1	D538G
		FGF19	amplification
		FGF3	amplification
		FGF4	amplification
		MYST3	amplification
		ZNF703	amplification

61	lymph node	ATM	E770*
		CDKN2A	loss
		CDKN2B	loss
		ARFRP1	amplification
		ESR1	amplification
		GATA3	H424fs*23+
		NFKBIA	amplification
		NKX2-1	amplification
		RPTOR	amplification
		ZNF217	amplification
62	liver	BRAF	G469A
		PTEN	loss
		TP53	R273C
		ARID1A	Q878*
63	liver	NF1	splice site 205_247del42
		BRIP1	N196S
		GATA3	A333FS*20
64	skin	PTEN	T319fs*6
		TP53	P98fs*54
		MYCL1	amplification
		CCND1	amplification
		JUN	amplification
		FGF19	amplification
		FGF3	amplification
		FGF4	amplification
		MLL2	P1131L
65	soft tissue	PIK3CA	E542K
		FGFR1	amplification
		FGFR2	P253R, Y375C
		MITF	V99I
		MYC	amplification
		MYST3	amplification
		ZNF703	amplification
66	liver	CCND1	amplification
		MEN1	I85fs*33
		ESR1	D538G
		FGF19	amplification
		FGF3	amplification
		FGF4	amplification
		GATA3	H292fs*8
		ZNF703	amplification
67	lymph node	PALB2	Q60fs*7, Y408fs*2
68	lymph node	PIK3CA	H1047L
		RUNX1	Q415fs*66+
		CDH1	Q346*
		ESR1	D538G
69	skin	PIK3CA	N345K

		CCND1	amplification
		ESR1	Y537S
		FGF19	amplification
		FGF3	amplification
		FGF4	amplification
		MSH2	E529*
70	breast	PTEN	C83fs*18
		CCND1	amplification
		FGFR1	amplification
		MYC	amplification
		CDH1	D587fs*2
		EMSY	amplification
		ESR1	D538G
		FGF19	amplification
		FGF3	amplification
		FGF4	amplification
		MYST3	amplification
		ZNF703	amplification
71	liver	PIK3CA	*1069fs*1+
		CCND1	amplification
		SRC	amplification
		ATR	ATR-XRCC4 fusion
		AURKA	amplification
		FGF19	amplification
		FGF4	amplification
		GNAS	amplification
		MDM2	amplification
		MYC	amplification
		TOP1	amplification
		ESR1	D538G
		FGF3	amplification
		ZNF217	amplification
72	soft tissue	PIK3CA	amplification
		CCND1	amplification
		KDR	amplification
		FGF19	amplification
		IGF1R	amplification
		NOTCH1	L2457V
		SOX2	amplification
		TP53	P151R
		ARFRP1	amplification
		ARID2	E71*, S1491*
		EMSY	amplification
		ESR1	Y537C
		FGF3	amplification
		FGF4	amplification
		GATA3	S411fs*29+

		MLL2	rearrangement exon 35
		ZNF703	amplification
73	skin	PIK3CA	H1047R
		CDH1	R209fs*6
		EMSY	R840H
		MAP3K1	Q1220*, splice site 2370-2_2381del14
74	unknown	PIK3CA	amplification, E542K
		MYC	amplification
		TP53	P151R
75	ovary	AUKRA	amplification
		GATA3	P409fs*38+
		ZNF217	amplification
